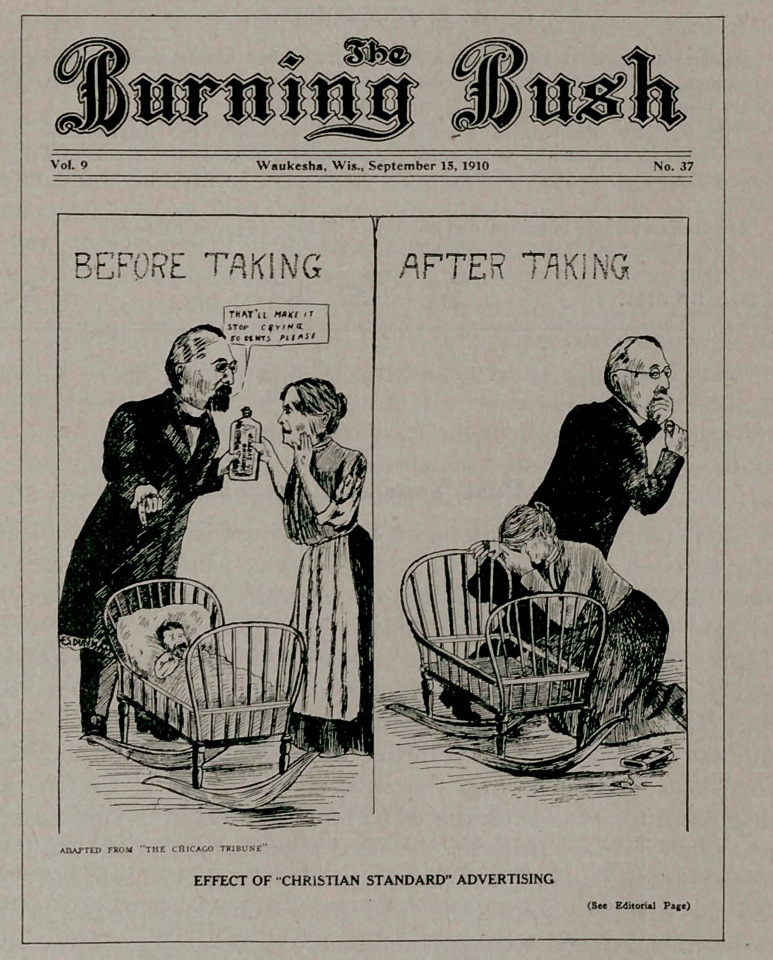# Our Contemporaries

**Published:** 1912-08

**Authors:** 


### OUR CONTEMPORARIES

---

### Getting Results From Prescribing

(Abstract from N. A. R, D. Propaganda )

The two legal standards, the United States Pharmacopœia and the National Formulary, contain some fifteen hundred dependable preparations and for that reason should represent the greater part of the medical profession's armamentarium at the present time. Every capable pharmacist will stand sponsor for their quality, uniformity and activity, without exception.

The physician should be absolute master of his prescription at all times, therefore it is suggested that all prescriptions containing narcotic or habit-forming drugs, be marked "Do not repeat, or "non-repetatur." A word of explanation to the patient as to the reason therefor would be proper.

Through prescribing by the official titles or Latin names only, the great harm caused by self-medication will receive a severe setback, and the physician will add to his own professional standing, and incidently promote the interests of his patients. Further, we believe that it is as much to the physician's interests to specify "those pharmacists who properly conduct their pharmarcies," as it is to specify "an ethical preparation." [Note our advertisers.]

Many owners and clerks in department stores and in some cut-rate drug stores who have a greater leaning toward the almighty dollar than toward honor, receive substantial commissions from the makers of many special lines of remedies, to boost and even substitute the latter products, contrary to the physician's wishes and implied instructions. Therefore it would be considered good judgment on the physician's part to exercise his prerogative in informing the patient where *not* to have his prescriptions filled.

There follows a detailed description of Pilulæ Ferri Carbonatis, U. S. P., and of Elixir Taraxaci Comp. N. F.

### Physicians' Investments

The *Physicions' Business Journal* is defunct but its spirit lives.

The *Clinical Reporter says:* "So keep your books that when a patient asks for his account you may render it with promptness and joy, not with trembling and hesitation."

"If you by any chance get a few dollars more than you need to pay expenses do not pass it over to the smooth tongued promoter. Trained speculators are looking for 'good things,' and do not allow big profit schemes to go a begging."

The *Medical Council* devotes a whole section to the Business Side of Practice, including articles on Irresponsible Pecan Promoters, Delusive Land Promoters, Personal Experience in Stock Investments besides two or three items which ought to elecit libel suits if the firms mentioned are not criminally liable.

One of the western states (The exact reference has been mislaid) has what is popularly termed "The Blue Sky Law" in allusion to the fact that it is aimed against promoters who are selling nothing more tangible. This law requires a license to sell stock and only a few of a large number of corporations applying have been given licenses, the remainder having been shown to be fake concerns. Yet one would suppose that no application would be made unless some evidence of good faith could be shown.

One of the writers in the *Council* reports excellent results from a conservative, local, investment in real estate in which he vainly tried to interest some medical friends who preferred slips of paper called stock.

We are glad that the medical journals are taking up this matter. It is high time doctors ceased to be classed as easy marks.

---

### If a Man is Wise He Will Not Work at Home, But Elsewhere

In an article on "The Man of the House" in the March *Woman's Home Companion,* the author, Margaret E. Sangster, says:

"The man of the house should not be the man in the house all day long, if it can be helped. If he be an artist with a studio at home, a minister with a study at the top of the house, a physician with office hours, or any other man who is accustomed to carry on his work at home, the household accommodates itself to him, and in a sense ignores his presence. It is true that he is constantly subjected to interruptions when he works at home, his wife softly opening the door to say, 'The milkman has called for his bill, and cannot make change,' or, 'Somebody has asked for you at the door. Will you not go down and see him?' or, 'Do pardon me for breaking in upon your work, but Aunt Jennie has just telephoned that she is coming to luncheon. Won't you step over to the butcher's and get a porterhouse steak?'

"It is not quite ideal for the man of the house to do his work at home. For his personal convenience and comfort it is preferable that he should do it elsewhere. The temptation to run in upon him, to read a letter, tell a bit of news, or ask advice, is too great for the average woman to overcome.

"Also, he gains something in the estimation of the family by going away in the morning and returning at night, while for himself there is the manifest advantage of a charming and restful change of scene when he steps within his front door to be stormed upon with kisses by the children and welcomed by his true comrade and partner on the road."

All of which is very true and somewhat unfortunate for the average physician who can not conveniently manage an office apart from his residence.

---

*American Medicine,* June, 1912, announces that its gold medal for " the most conspicuous and noteworthy service for humanity, in the domain of medicine during the past year," by an American physician, has been conferred upon Col. William Crawford Gorgas, Chief Sanitary Official of the Canal Zone.

The same journal deplores the small attendance at the A. M. A. meeting, appeals to the profession to take a more active interest, and offers the friendly criticism that such interest might be forthcoming if the profession generally had some part in the management of affairs. In particular, it suggests that the President should be more than a figure head and should, at least, have the veto power.

---

We acknowledge the courtesy of *American Medicine* in abstracting our article on the Economics of Food from the *Interstate Medical Journal* of April, 1912.

---

The *St. Louis Medical Review,* June, 1912, reprints our article on Wild Parsnip Poisoning, from the April issue.

---

*Merck's Archives,* June, 1912, reprints Col. Floyd S. Crego's article on Meningitis, from our March issue.

---

The *Medical Standard,* July, 1912, contains a full abstract of Dr. A. H. Noehren's article on Traumatic Finger Amputations, from our June issue.

---

The *Dominion Medical Monthly* comments as follows:

The National Insurance scheme of Mr. Lloyd George has now been before the English public for one year, yet success for the measure is not in sight.

That the medical profession is not treated fairly by this measure there are many evidences other than those set forth in the medical press.

The new move by the profession, designed to bring Mr. Lloyd George to his senses, is for the doctors to cut off all their contract relations with friendly societies.

It seems that the profession mistrust the insurance committees of the friendly societies, for on these committees the doctors will be in the minority.

The British Medical Association, through its State Sickness Insurance Committee, is now pledging members to resign from club and lodge practice and not to accept appointments under this Act.

---

The accompanying picture is taken from the *Burning Bush*, one of the batteries of the church militant. The *Burning Bush*

The Burning Bush

Vol. 9 | Waukesha, Wis., September 15, 1910 | No. 37

ADAPTED FROM "THE CHICAGO TRIBUNE"

EFFECT OF "CHRISTIAN STANDARD" ADVERTISING

(See Editorial Page)

believes in some things that the average professional man does not, and vice versa but it stands for sincerity. It hits hard at the hypocrisy of an orthodox church passing over to liberalism or altering its time-honored standards to insure popularity among the young folks. The meanest part of such hypocrisy is that it undermines and seeks to steal the proper reward of the heterodox organizations which, half a century or more ago, suffered the persecutions of the orthodox. But our readers will be most interested in the campaign of the *Burning Bush* against the prostitution of religious papers in advertising nostrums.

## Figures and Tables

**Figure f1:**